# Implantation of a Cushioning Injectable Implant Using Needle Arthroscopy in the Foot and Ankle and First Carpometacarpal Joint

**DOI:** 10.1016/j.eats.2023.08.004

**Published:** 2023-11-27

**Authors:** Tobias Stornebrink, Alex Walinga, Miki Dalmau-Pastor, Anton W. Bosman, Theodoor H. Smit, Gino M.M.J. Kerkhoffs

**Affiliations:** a Department of Orthopedic Surgery and Sports Medicine, Amsterdam UMC location University of Amsterdam, Amsterdam, the Netherlands; b Amsterdam Movement Sciences, Amsterdam, the Netherlands; c Amsterdam Collaboration for Health & Safety in Sports (ACHSS), International Olympic Committee (IOC) Research Center Amsterdam UMC, Amsterdam, the Netherlands; d Human Anatomy and Embryology Unit, Department of Pathology and Experimental Therapeutics, School of Medicine and Health Sciences, University of Barcelona, Barcelona, Spain; e MIFAS by GRECMIP (Minimally Invasive Foot and Ankle Society), Merignac, France; f SupraPolix BV, Horsten 1, Eindhoven, the Netherlands

## Abstract

Injectable implants constitute a newly developed treatment class in the battle against osteoarthritis. They consist of water-formulated supramolecular polymer, coming from a new class of resorbable biomedical materials, and are implanted in encapsulated joints in a liquid form, where they solidify to form a tough, elastic, and cushioning layer between the joint surfaces. To resort any effect, intra-articular delivery should be guaranteed, and the implant should be distributed throughout the entire joint space. Traditional implantation techniques do not seem to suffice for this new implant class, being either imprecise (traditional injection) or overly invasive (open procedures and traditional arthroscopic surgery). We describe a needle arthroscopic implantation technique to reap the benefits of both worlds, ensuring precise implant delivery while avoiding unnecessarily invasive procedures. This study depicts our needle arthroscopic technique for implantation of injectable implants in the ankle, first metatarsophalangeal joint, and first carpometacarpal joint.

Osteoarthritis is a multifactorial disease that involves synovial inflammation and degeneration of the cartilage matrix. Mechanical trauma forms the onset of the disease in a large percentage of patients,^1^ as this trauma causes radiologically invisible but mechanically measurable bruises in articular cartilage.^2^ Recent studies in a rat model (2021) showed that posttraumatic offloading suppresses the development of osteoarthritis.^3^ In contrast, complete unloading results in disuse atrophy of cartilage, implying a tradeoff between loading and unloading of cartilage.^4^

We describe a material that functions as an internal shock absorber and acts as a protective joint cushion. It dampens mechanical loading of articular cartilage but does not result in complete unloading of the joint.^5^ The material can be injected in its liquid form and aims to fill the entire joint space. It subsequently solidifies within minutes to form a tough, elastic, and cushioning layer between the joint surfaces. It aims to reduce shear stress on the articular cartilage and restore hydrostatic pressure, thereby shifting the state of chondrocytes from katabolic and inflammatory to anabolic and regenerative. This induces an optimal mechanical environment, which should (1) protect cartilage in high-risk situations such as high-demand sports, (2) prevent further cartilage damage after initial focal bruising, or (3) reduce symptoms in advanced-stage cartilage deterioration.

The injectable implant should be delivered in a well-encapsulated joint space, and distribution throughout the entire joint space should be ensured. Conventional injection techniques that are guided by palpation or various imaging modalities may result in an unacceptably high chance of extra-articular delivery of the implant,^6^^,^^7^ yet open or traditional arthroscopic surgery is highly invasive and seems to go against the simple nature of the joint cushion approach. We therefore describe a needle arthroscopic implantation technique in order to ensure correct implant positioning and preserving the minimally invasive nature of the joint cushion.

The current paper describes our technique for implantation of a cushioning injectable implant using needle arthroscopy. Implantation in 3 joints is described: the ankle (talocrural), first metatarsophalangeal joint (MTP-1), and first carpometacarpal joint (CMC-1).

## Surgical Technique (With Video Illustration)

Video 1 provides a step-by-step demonstration of the technique. Cadaveric specimens used to display the technique were donated with consent for use in medical science and obtained through the donation programs of the Human Anatomy and Embryology Unit of the University of Barcelona and the Amsterdam University Medical Centers. The study was conducted in agreement with the 1964 Helsinki Declaration and its later amendments. Ethical approval by our institution’s review board was not required.

### Equipment

We use a 1.9-mm diameter disposable needle arthroscope (NanoScope; Arthrex, Naples, FL). This needle arthroscope is semirigid, has a 0° viewing angle, and a 100° field of vision. It is connected to a tablet-like console that processes, stores, and shows all imaging. The disposable kit contains a blunt and a sharp obturator, and a 2.2-mm diameter cannula. A standard needle and syringe can be used through a second injection portal.

### Injectable implant

We used an injectable cushioning implant (n-ICE; Injectable Implants, Amsterdam, The Netherlands), which is based on supramolecular polymers as developed by SupraPolix BV (Eindhoven, The Netherlands). The used materials are water-based injectable liquids that solidify into a highly compressible material within minutes when injected.^8^

### Anesthesia

The surgical field is disinfected, and standard surgical draping is applied. Lidocaine 2% is used to anesthetize the arthroscopic portal along its entire tract from skin to joint capsule. There is no need to anesthetize the second injection portal. Sedation is not required.

### Ankle (Talocrural)

**Fig 1:**
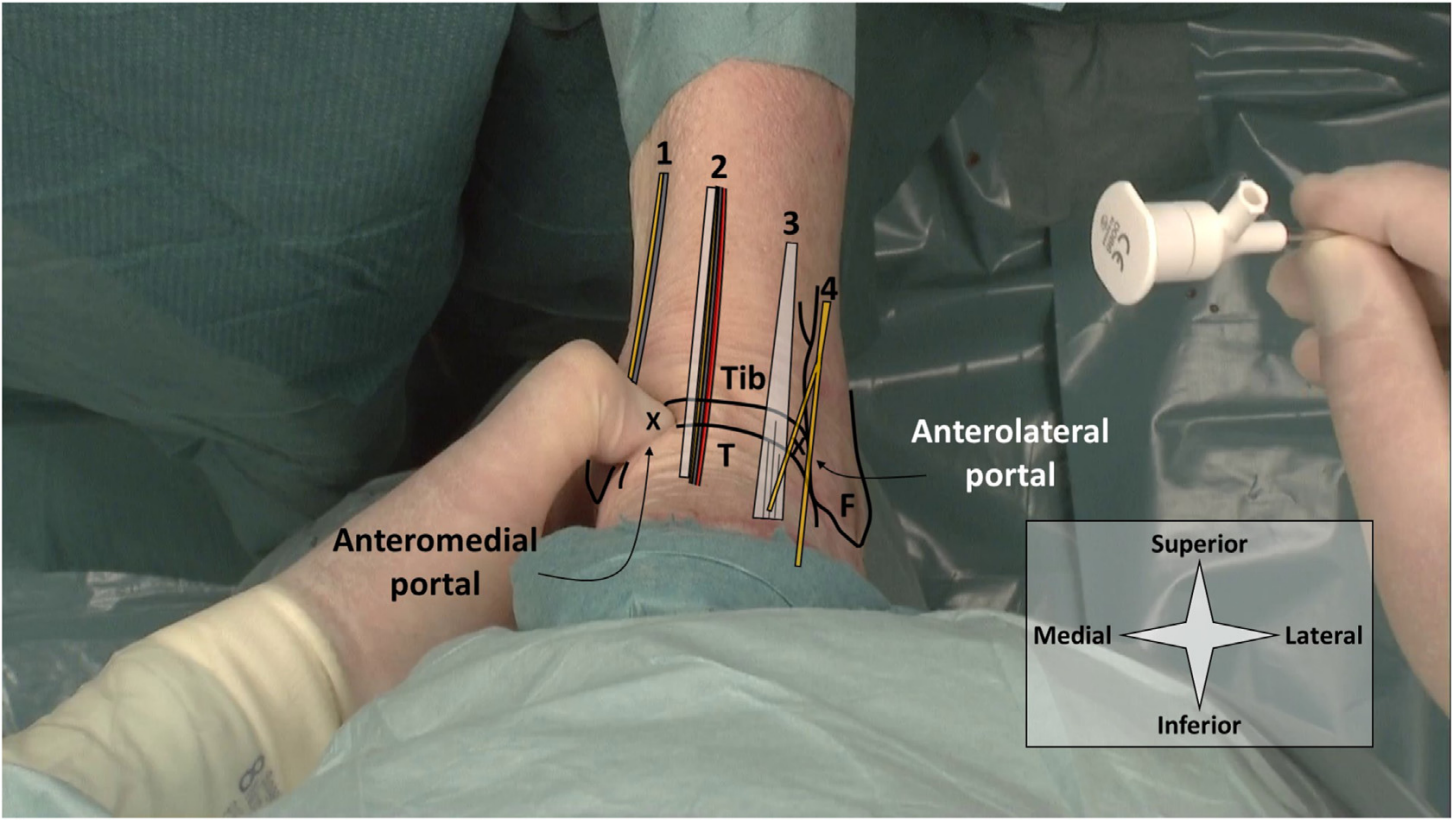
Shown are portal locations for needle arthroscopic delivery of injectable implants in the ankle (talocrural). A left ankle is seen from a top-down surgeon perspective with the patient in a supine position. Anatomic landmarks and structures at risk are depicted schematically. The surgeon’s thumb palpates the anteromedial soft spot. Proper anesthesia of the entire portal tracts is ensured, including the joint capsule. To avoid iatrogenic cartilage damage during portal placement, the ankle is kept in dorsiflexion. The x’s denote the respective portal locations. Tib denotes the tibia, T the talus, and F the fibula. From medial to lateral, 1 contains the saphenous nerve and great saphenous vein, 2 the anterior tibial tendon and anterior neurovascular bundle, 3 the extensor digitorum longus tendon, and 4 the superficial peroneal nerve with its medial dorsal cutaneous branch (superior) and intermediate dorsal cutaneous branch (inferior).

The patient is positioned supine with their heels at the edge of the bed or operating table. An anteromedial and an anterolateral portal are used and identified by palpation (Fig 1). With the ankle in dorsiflexion, the anteromedial portal is located on the soft spot medial to the anterior tibial tendon and at the anterior joint line.^9^ The anterolateral portal is located lateral to the peroneus tertius tendon (or to the extensor digitorum longus in case of absence of the tertius), again at the anterior joint line.^9^

**Fig 2:**
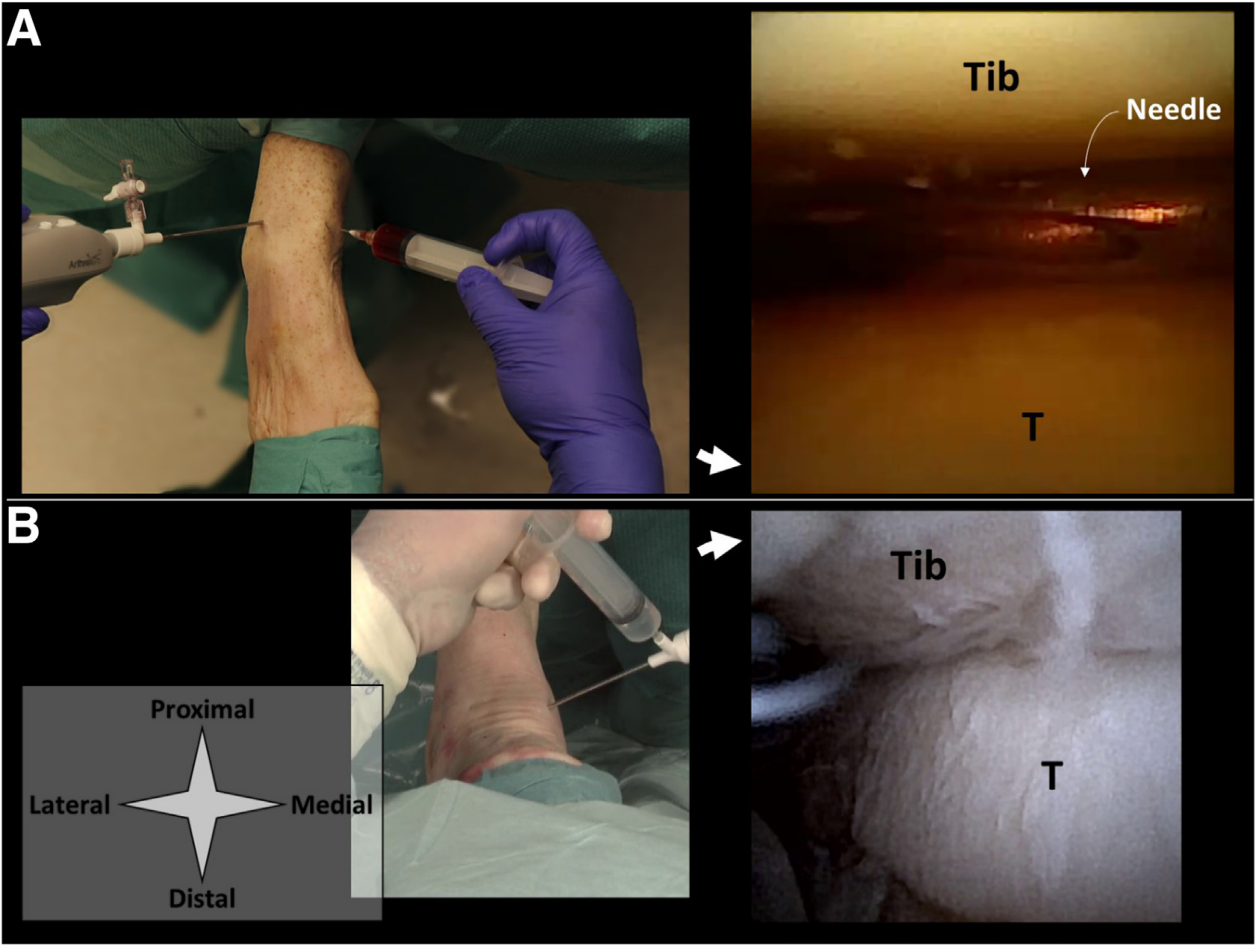
Shown is the delivery of an injectable implant in the ankle. A right ankle in supine position is seen from a top-down surgeon’s perspective, with corresponding intra-articular views from the needle arthroscope. Injections can be delivered either by guiding a second syringe and needle to a specific location within the joint (A), or directly through the needle arthroscope with intra-articular positioning confirmed on the arthroscopic view (B). The injectable implant is delivered with a second syringe as depicted in option (A). The arthroscopic view can be used to confirm intra-articular positioning and ensure distribution through the joint. Fluids with lower viscosity can alternatively be delivered through the arthroscopic cannula itself (B), which eliminates the need for a second portal. (T, talus; Tib, tibia.)

The anteromedial portal is created first. The skin is prepared with a 2-mm stab incision. A 2.2-mm diameter cannula is then loaded with a blunt obturator and the cannula is penetrated through the joint capsule and entered intra-articular. During portal placement the ankle is kept in dorsiflexion to protect weight-bearing cartilage. Slight noninvasive distraction may be of help in achieving intra-articular positioning. The obturator is removed from the cannula and replaced with the 1.9-mm diameter needle arthroscope. The anterolateral portal can now be established under intra-articular visualization (Fig 2).

### MTP-1

**Fig 3:**
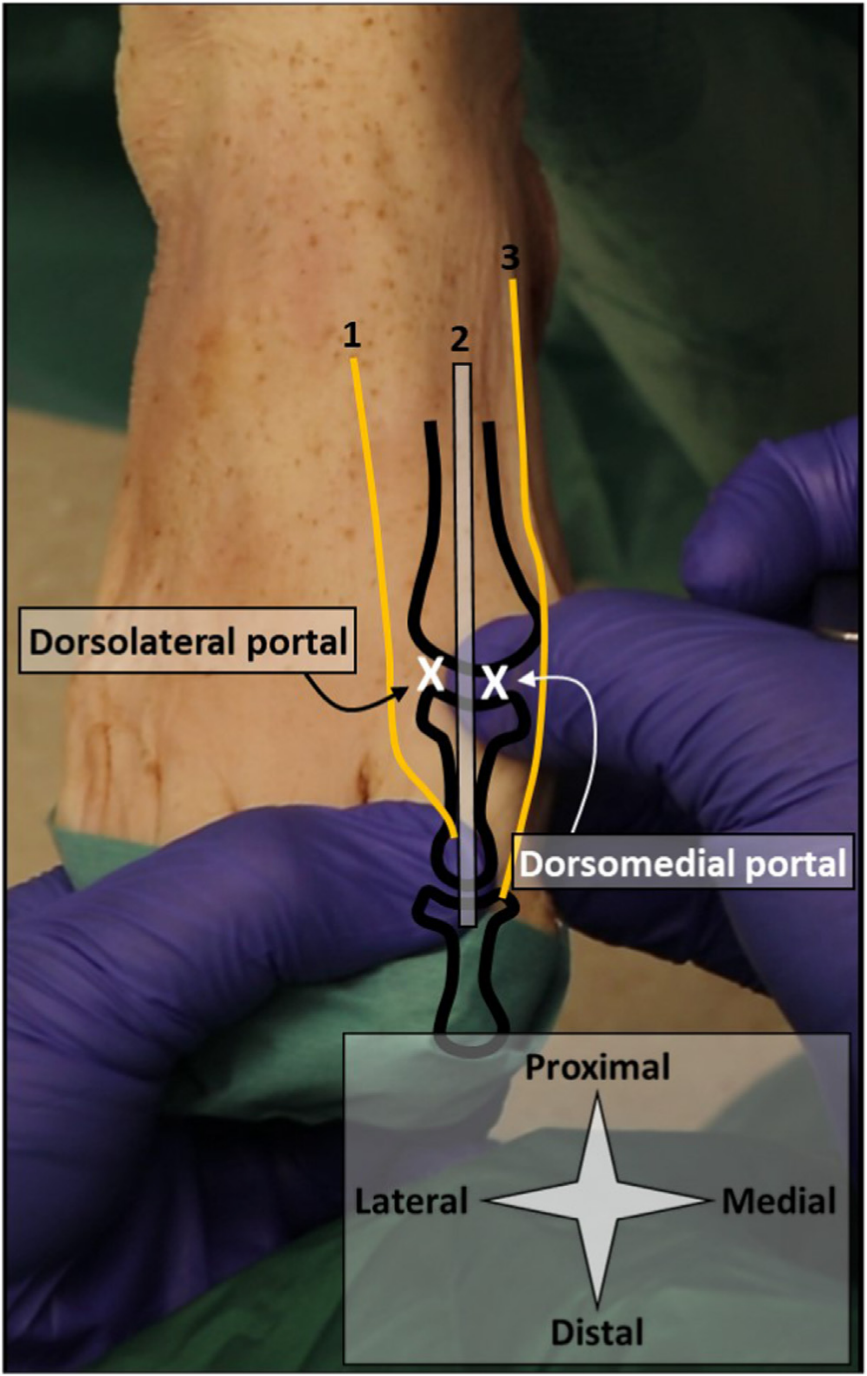
Shown are portal locations for needle arthroscopic delivery of injectable implants in the MTP-1 joint. A right foot is seen from a top-down surgeon perspective with the foot in supine position and slightly plantar flexed in the ankle. Anatomic landmarks and structures at risk are depicted schematically. The surgeon’s left hand pulls the proximal phalanx in the distal direction, distracting the MTP-1 joint. This creates a sulcus at the level of the dorsomedial portal, which is palpated by the surgeon’s right thumb. The x’s denote the respective portal locations. The dorsolateral portal is used for needle arthroscopy. This portal is created with a 2-mm stab incision of the skin and subsequent blunt introduction of the needle arthroscopic cannula. Make sure to properly anesthetize this portal, from skin to joint capsule. 1 denotes the dorsolateral hallucal nerve, 2 denotes the extensor hallucis longus tendon, and 3 denotes the dorsomedial hallucal nerve. (MTP-1, first metatarsophalangeal joint.)

**Fig 4:**
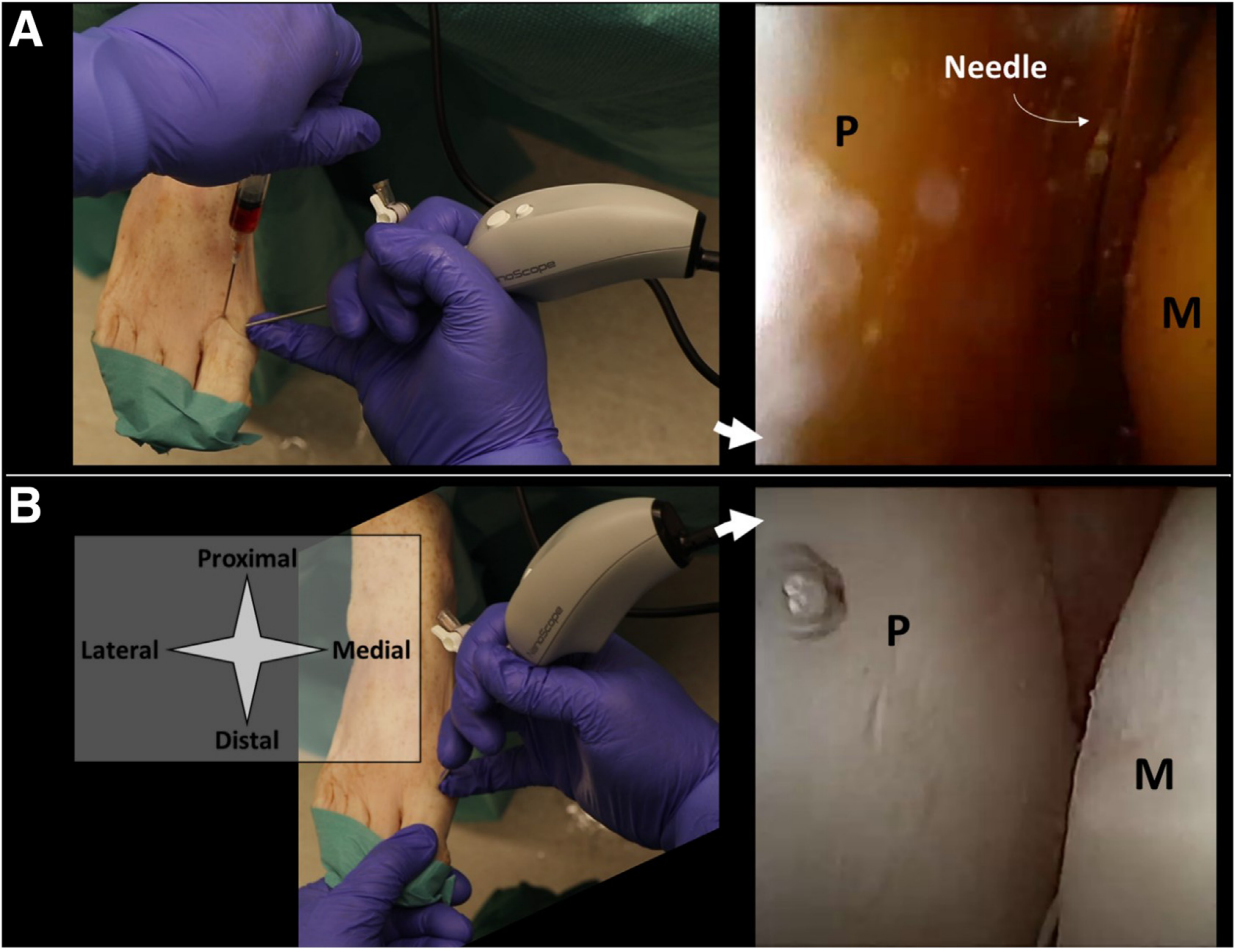
Shown is the delivery of an injectable implant in the MTP-1 joint. A right foot is seen from a top-down surgeon perspective with the foot in supine position and slightly plantar flexed in the ankle, with corresponding intra-articular views from the needle arthroscope. Injections can be delivered either by guiding a second syringe and needle to a specific location within the joint (A), or directly through the needle arthroscope with intra-articular positioning confirmed on the arthroscopic view (B). The injectable implant is delivered with a second syringe as depicted in option a. The arthroscopic view can be used to confirm intra-articular positioning and ensure distribution through the joint. Fluids with lower viscosity can alternatively be delivered through the arthroscopic cannula itself (B), which eliminates the need for a second portal. P denotes the base of the first proximal phalanx, M the tip of the first metatarsal bone. (MTP-1, first metatarsophalangeal joint.)

The patient is positioned supine with their heels at the edge of the bed or operating table. A dorsomedial and dorsolateral portal are used and identified by palpation (Fig 3). Manually, the proximal and distal phalanx are pulled in distal direction to distract the toe at the level of the MTP-1 joint. This distraction creates a sulcus at the dorsal side of the MTP-1 joint, facilitating its identification. The dorsomedial and dorsolateral portals are located just medial and lateral to the extensor hallucis longus tendon, respectively. The dorsolateral portal is used for needle arthroscopy and created with a 2-mm stab incision of the skin and subsequent blunt penetration of the joint capsule using the cannula and blunt obturator. The dorsomedial portal can now be established under intra-articular visualization (Fig 4).

### CMC-1

**Fig 5:**
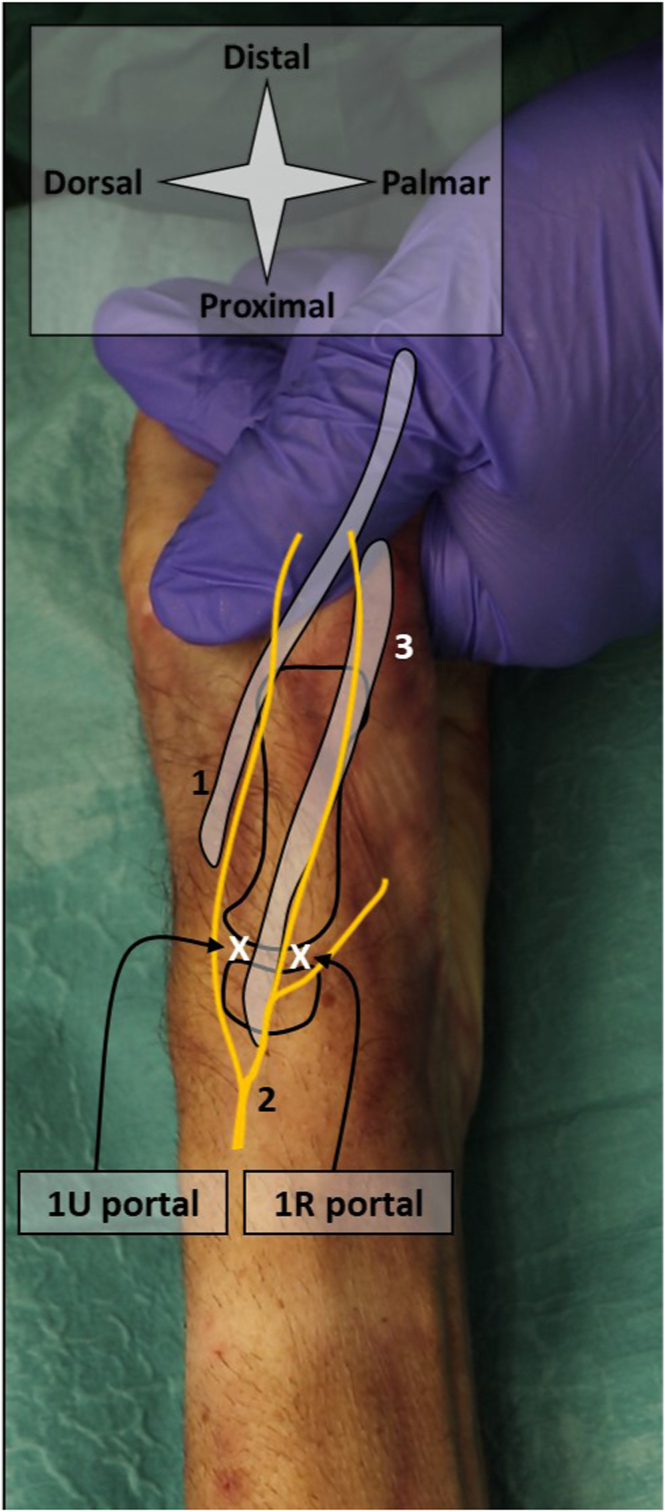
Shown are the portal locations for needle arthroscopic delivery of injectable implants in the CMC-1 joint. Anatomic landmarks and structures at risk are depicted schematically on a right hand. The surgeon’s hand pulls the thumb distally, applying distraction of the CMC-1 joint. The 1U portal is used for needle arthroscopy. This portal is created with a 2-mm stab incision of the skin and subsequent blunt penetration of the needle arthroscopic cannula. Make sure to properly anesthetize this portal, from skin to joint capsule. The x’s denote the respective portal locations. 1 denotes the extensor pollices longus tendon; 2 denotes the radial nerve and its branches; and 3 denotes the extensor pollices brevis tendon. (CMC-1, first carpometacarpal.)

**Fig 6:**
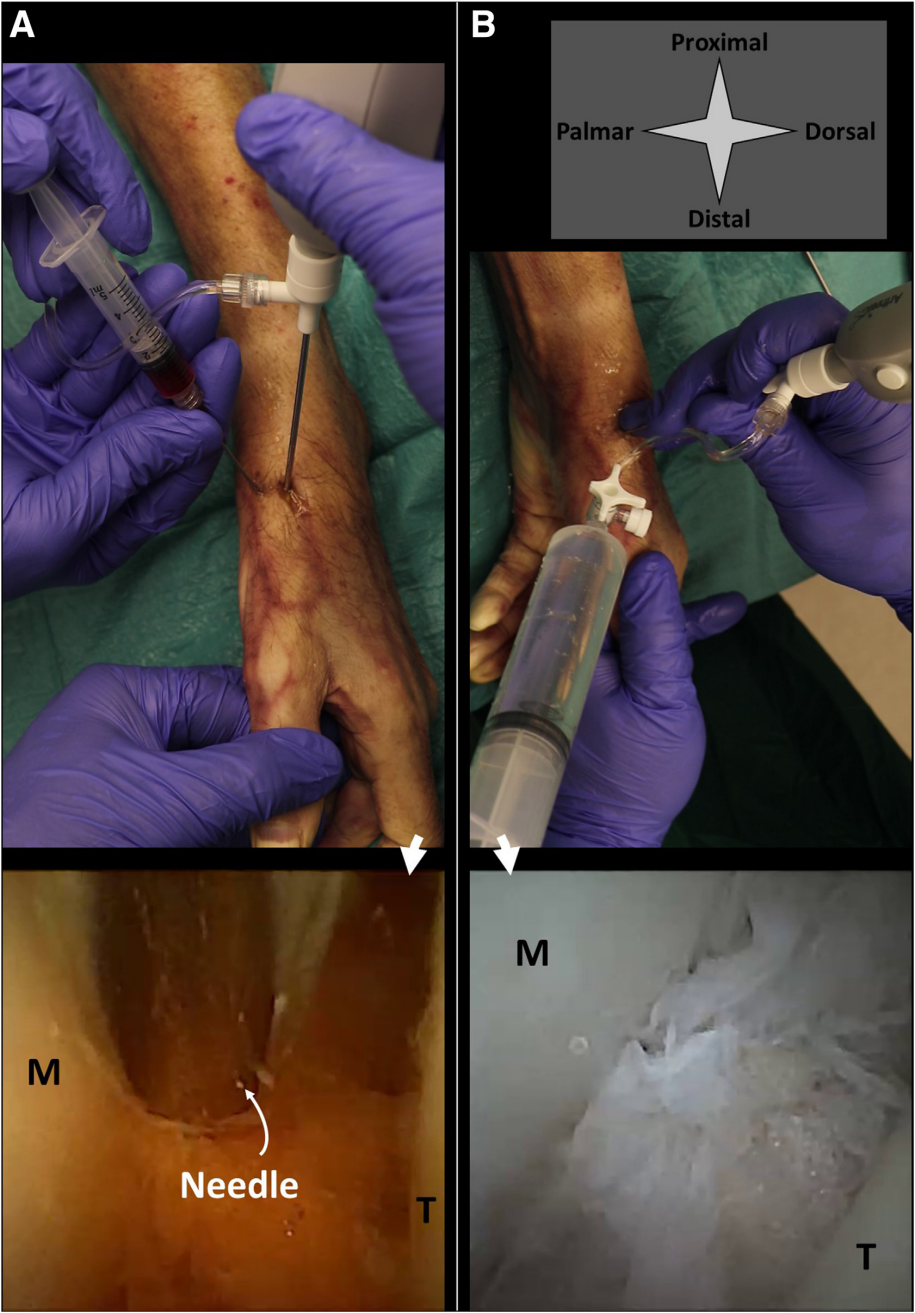
The delivery of an injectable implant in the CMC-1 joint. A left hand is seen from a top-down surgeon perspective, with corresponding intra-articular views from the needle arthroscope. Injections can be delivered either by guiding a second syringe and needle to a specific location within the joint (A), or directly through the needle arthroscope with intra-articular positioning confirmed on the arthroscopic view (B). The injectable implant is delivered with a second syringe as depicted in option a. The arthroscopic view can be used to confirm intra-articular positioning and ensure distribution through the joint. Fluids with lower viscosity can alternatively be delivered through the arthroscopic cannula itself (B), which eliminates the need for a second portal. M denotes the base of the first metacarpal bone, T the trapezium bone. (CMC-1, first carpometacarpal.)

The patient is positioned in a sitting position, with their arm resting on an arm table. The 1R and 1U portals are used (Fig 5) and identified by palpation. Manually, the thumb is pulled in distal direction in order to distract the finger at the level of the CMC-1 joint. The 1U portal is used for needle arthroscopy and located just ulnar to the extensor pollicis brevis tendon. It is created with a 2-mm stab incision of the skin and subsequent blunt penetration of the joint capsule using the cannula and blunt obturator. The 1R portal can now be created under intra-articular visualization. The 1R portal is located just radial to the abductor pollicis longus tendon (Fig 6).

### Inspection, Injection, and Closure

A diagnostic inspection can be performed after introduction of the needle arthroscope. Once intra-articular positioning is confirmed on the arthroscopic view, injections may be performed by inserting a second needle under needle arthroscopic guidance (Figs 2A, 4A, 6A). The arthroscopic view can be used to ensure equal distribution throughout the joint space. Once the procedure is finished, all equipment is removed and the portals are closed with sterile wound closure strips. In case of implants with lower viscosity, or in case water distention is needed for arthroscopic inspection prior to implant delivery, these fluids can be directly injected through the arthroscopic cannula itself as well, as is depicted in Figures 2B, 4B, and 6B.

### Hardening

**Fig 7:**
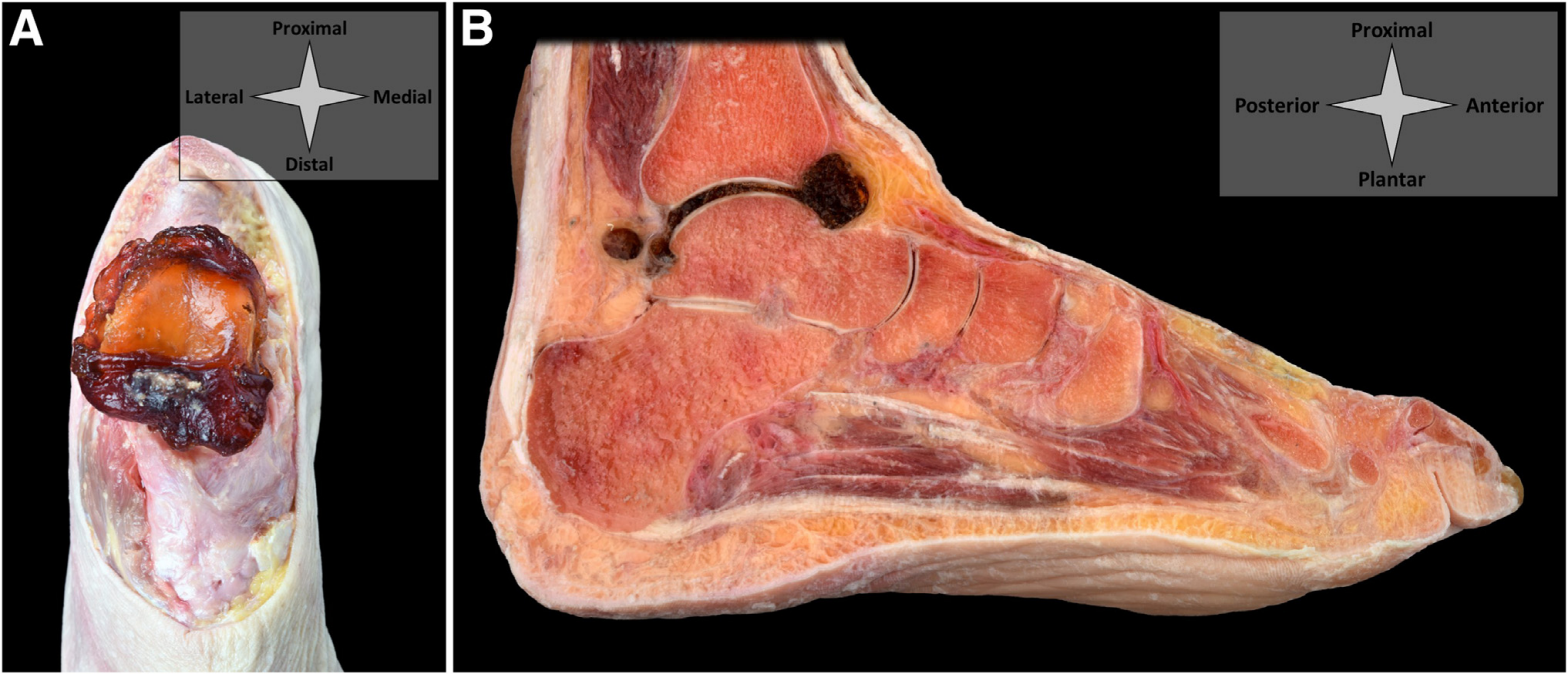
Shown is a cushioning implant in its solid state upon needle arthroscopic injection in the talocrural joint. In (A), the ankle is seen from a top-down perspective and disarticulated at the level of the talocrural articulation. In (B), a sagittal cut is shown. The dissections show that the implant has spread throughout the entire joint and is contained within the joint capsule. It has formed a tough, elastic and cushioning layer between the joint surfaces.

Upon injection, patients are left to rest non–weight-bearing in order to allow the injected cushion to harden and form a solid implant. The implant as used for this demonstration requires a 10-minute rest upon injection and reaches its maximum stiffness after 8 hours. Figures 7 (ankle) and 8 (MTP-1 and CMC-1) demonstrate the injected implant in its solid form in cadaveric specimens.

**Fig 8:**
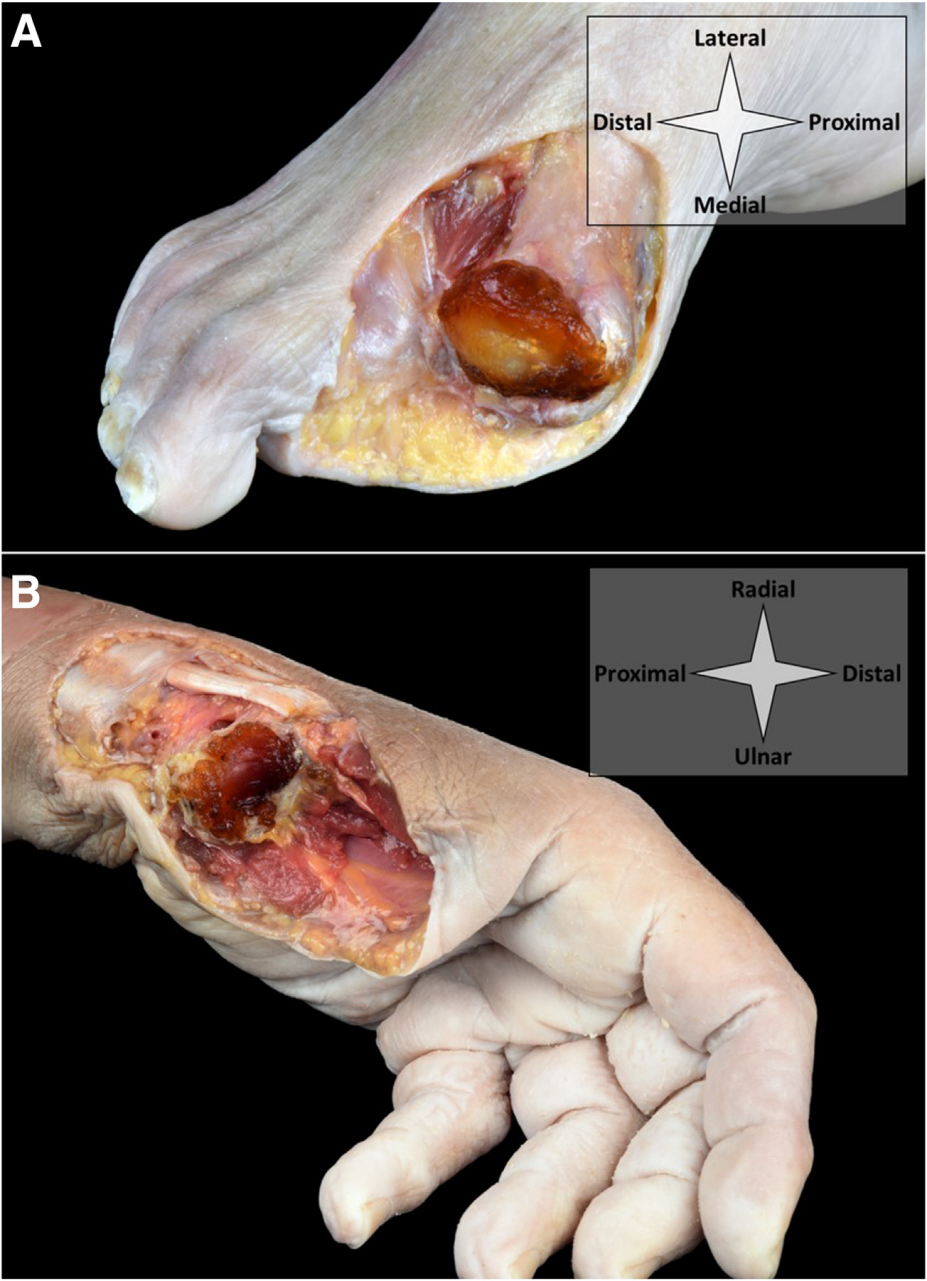
Shown is a cushioning implant in its solid state upon needle arthroscopic injection in the MTP-1 (A) and CMC-1 (B) joints. For demonstration, the joints have been disarticulated at the level of MTP-1 (A) and CMC-1 (B) and dissected up to and including the joint capsule. The implant has spread throughout the entire joint and has been contained within the joint capsule. It has formed a tough, elastic and cushioning layer between the joint surfaces. (CMC-1, first carpometacarpal; MTP-1, first metatarsophalangeal joint.)

## Discussion

The joint cushion described here constitutes a novel treatment class: injectable implants. It provides partial and temporary internal distraction, restores hydrostatic pressure, and can be combined with other soluble therapeutics to form a slow-releasing drug-delivery system. Human cadaveric studies demonstrate that such an injectable cushion is particularly suited for the ankle and MTP-1 and CMC-1 joints, as depicted in this technique study as well. The ankle and MTP-1 and CMC-1 joints are well-encapsulated joints, which ensures containment of the injectable implant. In addition, a novel treatment class seems of great added value here, as these are joints with high prevalence of osteoarthritis, yet lack satisfying traditional treatment options.

Needle arthroscopy has already been used in clinical practice to guide articular injections. These injections were well-tolerable for patients under local anesthesia and the needle arthroscopic guidance resulted in high injection accuracy.^10^ However, when considering needle arthroscopy to guide intra-articular injections, surgeons should be aware of potential pitfalls (Table 1). Compared with standard injection needles, needle arthroscopy does create a slightly larger portal diameter, and when needle arthroscopy is used to guide a second needle, it evidently necessitates a second portal. In the ankle, it has been shown that gaining needle arthroscopic access to the joint can be difficult in last-stage osteoarthritis, and in case of soft-tissue that is cicatrized by previous surgery.^10^ One should hence carefully scrutinize their indication for needle arthroscopic guidance of injections. Nonetheless, previous studies do support the safety of in-office needle arthroscopic procedures,^11^^,^^12^ and various techniques have been described.^13^, ^14^, ^15^, ^16^, ^17^ Pearls of the procedure are listed in Table 1.

**Table 1:** Pearls and Pitfalls

Pearls	Pitfalls
Intra-articular positioning can be ensured and the procedure can be combined with an arthroscopic inspection	Needle arthroscopy requires a slightly larger portal diameter compared with standard injection needle
Needle arthroscopy ensures a minimally invasive procedure, whilst reaping benefits of arthroscopy	A second injection portal is required for implant delivery
Injectable implants constitute a new treatment class in the battle against osteoarthritis. They may induce an optimal mechanical environment, which should (1) protect cartilage in high-risk situations such as high-demand sports, (2) prevent further cartilage damage after initial focal bruising, or (3) reduce symptoms in advanced-stage cartilage deterioration.	The 0-degree direction of view may require a learning curve for those used to conventional arthroscopy
Injectable implants may be combined with soluble therapeutics to form a slow-releasing drug-delivery system.	Previous surgery and advanced-stage osteoarthritis may hamper introduction of the needle arthroscope
	Intra-articular positioning is paramount for the implant to resort any effect
	Large defects in the joint capsule may result in extra-articular dispersion of the implant

As-yet-unpublished animal studies showed feasibility and safety of the joint cushion, with no local or systemic adverse effects or inflammatory responses. In terms of risks (Table 1), these studies did show that discontinuities in the joint capsule—for example, in case of an intra-articular trajectory of a large tendon or after extensive surgery—can result in extra-articular spreading of the implant. These defects in the joint capsule have to be of a substantial size for extra-articular displacement to occur, as in no case there was dispersion through injection and needle arthroscopy portals. Although extra-articular displacement of the implant had no adverse effects on surrounding soft tissue in animal studies, it will likely reduce the therapeutic effect of the implant in the joint itself. Therefore, in situations in which such substantial defects may be present, implantation should be carefully performed and the indication should be well-advised. Furthermore, patients should be counseled that there may be a need for repeat injections upon implant degradation, and this need may be especially likely in patients suffering from late-stage osteoarthritis.

The injectable implant that was used for this demonstration requires a separate needle for delivery, as its viscosity is too high for implantation through the arthroscope itself. Implants or substances with lower viscosity could be delivered directly through the arthroscopic cannula—as is depicted in this paper as well—eliminating the need for a second portal. This 2-portal setup does provide an effective means to rinse the joint and clear it from debris, synovial fluid and inflammatory products, as already described elsewhere.^16^ However, for injectable implants it is not required to rinse the joint, and notwithstanding the presence of synovial fluid, the injectable implant showed good solidification upon injection.

In conclusion, this study provides a stepwise approach to needle arthroscopically guided implantation of a cushioning injectable implant in the ankle, MTP-1 and CMC-1 joints, allowing a possible novel treatment for osteoarthritis. The needle arthroscopic technique aids to accurately deliver this cushion or other injectables in these joints and ensures distribution throughout the joint or to local areas of interest. It can be performed under local anesthesia and combined with an arthroscopic inspection.

## Supplementary Data

ICMJE author disclosure forms

Video 1 Shown is a step-by-step demonstration of implantation of a cushioning injectable implant using needle arthroscopy in the foot and ankle and CMC-1 joint. A short background regarding injectable implants is provided first, including the demonstration of an injection in a model joint. Cadaveric specimens are then used for demonstration, including a right ankle, right MTP-1 and left CMC-1. For each joint, portal placement is shown, followed by delivery of the implant through a second, standard injection needle. The implant is injected in its liquid form. Upon injection, patients are left to rest non–weight-bearing in order to allow the injected cushion to harden and form a solid implant. The implant as used for this demonstration requires a 10-minute rest upon injection and reaches its maximum stiffness after 8 hours. It then functions a tough, elastic and cushioning layer between the joint surfaces, aiming to reduce shear stress and restore hydrostatic pressure. (CMC-1, first carpometacarpal; MTP-1, first metatarsophalangeal joint.)
